# A New Approach to Retinal Oxygen Extraction Measurement Based on Laser Speckle Flowgraphy and Retinal Oximetry

**DOI:** 10.1167/tvst.13.12.12

**Published:** 2024-12-11

**Authors:** Viktoria Pai, Patrick Janku, Theresa Lindner, Ulrich Graf, Leopold Schmetterer, Gerhard Garhöfer, Doreen Schmidl

**Affiliations:** 1 Department of Clinical Pharmacology, Medical University of Vienna, Vienna, Austria; 2 Singapore Eye Research Institute, Singapore National Eye Centre, Singapore; 3 Ophthalmology and Visual Sciences Academic Clinical Program, Duke-NUS Medical School, Singapore; 4 SERI-NTU Advanced Ocular Engineering (STANCE), Nanyang Technological University, Singapore; 5 School of Chemistry, Chemical Engineering and Biotechnology, Nanyang Technological University, Singapore; 6 Center for Medical Physics and Biomedical Engineering, Medical University of Vienna, Vienna, Austria; 7 Fondation Ophtalmologique Adolphe De Rothschild, Paris, France

**Keywords:** retinal oxygen extraction, retinal blood flow, hyperoxia, hypoxia, reproducibility, laser speckle flowgraphy, LSFG, retinal oximetry

## Abstract

**Purpose:**

Currently, no standard for the measurement of retinal oxygen extraction exists. Here, we present a novel approach for measurement of retinal oxygen extraction based on two commercially available devices, namely laser speckle flowgraphy (LSFG) and retinal oximetry.

**Methods:**

The study was conducted in a randomized, double-masked design. Two study days were scheduled for each healthy participant. On one study day, measurements were performed during breathing of 100% oxygen to induce hyperoxia and on the other study day during breathing of 12% oxygen in nitrogen to induce hypoxia. To obtain data for short- and long-term reproducibility, baseline measurements during breathing of room air were performed twice on both study days. Retinal oxygen extraction was calculated from retinal oxygen saturation measurements using the oxygen module of the dynamic vessel analyzer (Imedos, Jena, Germany) and retinal blood flow measurements using LSFG (Nidek, Tokyo, Japan).

**Results:**

As expected, breathing of 100% oxygen induced a significant decrease in retinal oxygen extraction of 36% ± 17% (*P* < 0.001). During hypoxia, retinal oxygen extraction did not change from baseline (*P* = 0.153). For short-term reproducibility, the intraclass correlation coefficient was excellent (0.910) and good (0.879) for long-term reproducibility. Coefficient of variation between measurements was 9.8% ± 7.0% for short-term and 10.4% ± 8.8% for long-term reproducibility.

**Conclusions:**

The data obtained in the present experiments show that the new approach to measure retinal oxygen extraction is valid and reproducible in healthy volunteers.

**Translational Relevance:**

The technique may become a valuable tool in studying retinal hypoxia in a wide variety of ocular and systemic diseases in the future.

## Introduction

Adequate oxygen supply is crucial for retinal function, and alterations in retinal oxygen metabolism are associated with various ophthalmic, as well as systemic diseases such as glaucoma, age-related macular degeneration, diabetes, multiple sclerosis, or Alzheimer's disease.^1^^–^^8^ In recent years, different methods for the assessment of retinal oxygen metabolism have been proposed. One approach is to measure oxygen saturation in retinal arteries and veins and calculate the difference, which is often referred to as arteriovenous oxygen saturation difference.^9^^,^^10^ The advantage of this method is that it is quick and easy, but it does not take blood flow into account, as for example a decreased arteriovenous difference in oxygen saturation alone does not necessarily mean a decline in retinal oxygen extraction, because it could be compensated by an increase in retinal blood flow.^11^

Measurement of retinal oxygen extraction by combining measurement of total retinal blood flow and retinal oxygen saturation is an alternative approach that provides a more direct measure of retinal oxygenation status.^11^ Several methods have been proposed for the assessment of retinal blood flow.^10^^,^^12^ We have previously introduced a method to calculate retinal oxygen extraction based on total retinal blood flow measurements with Doppler optical coherence tomography (Doppler OCT) and retinal oxygen saturation obtained by retinal oximetry.^3^^,^^7^^,^^11^^,^^13^^,^^14^ Although this approach showed good reproducibility, a major disadvantage is that there is no commercially available Doppler OCT on the market.^15^ A recent publication, using a different custom-built device, suggested that measurement of retinal oxygen extraction could replace biochemical tests to predict nonproliferative diabetic retinopathy in the future.^16^

In this article, we introduce a new approach for measurement of retinal oxygen extraction based on retinal oximetry and laser speckle flowgraphy (LSFG). LSFG has several advantages compared to other techniques for measurement of retinal blood flow because measurements only take a few seconds, are highly reproducible and the location of previous measurements can exactly be found during follow-up examinations.^17^^,^^18^ LSFG has been successfully applied in a variety of patients and in studies investigating retinal physiology.^18^^–^^26^

To test our approach, we measured retinal oxygen extraction during breathing of 100% oxygen (hyperoxia) and during breathing of 88% nitrogen in 12% oxygen (hypoxia) in healthy subjects, because it is well established that retinal oxygen extraction decreases during hyperoxia but remains stable during hypoxia.^11^^,^^27^^,^^28^ Additionally, we also tested short- and long-term reproducibility of our approach in a subset of subjects.

## Methods

### Subjects

The study protocol was approved by the Ethics Committee of the Medical University of Vienna. The Declaration of Helsinki and Good Clinical Practice (GCP) guidelines of the European Union were followed throughout the duration of the study. Each subject provided written informed consent to participate in the study. Participants were recruited by the Department of Clinical Pharmacology at the Medical University of Vienna.

Before the first study day, a screening examination was carried out on each study participant, which included medical history, urine pregnancy test in women with childbearing potential (as required by Austrian Medicine Act in any clinical study), physical examination, 12-lead electrocardiogram, hemodynamic measurements and ophthalmological examination which included visual acuity, slit lamp biomicroscopy, indirect funduscopy and measurement of intraocular pressure (IOP) with Goldmann applanation tonometry.

Eligible patients were men and women aged 18 to 35 years, with normal ophthalmic findings, ametropia of 6 diopters or less, a normal medical history and physical examination (unless an abnormality was deemed clinically irrelevant by the investigator), and who were non-smokers.

The following criteria excluded subjects from the study: regular use of any medication (except contraceptives), abuse of alcoholic beverages or drugs, participation in a clinical trial in the 3 weeks preceding the study, treatment with any drug (except contraceptives) within 3 weeks prior to the study, symptoms of a clinically relevant illness in the 3 weeks before the first study day, blood donation during the previous 3 weeks before the study, pregnant or breastfeeding women and women of childbearing potential not using effective contraception.

### Study Design

The study was conducted in a randomized, double-masked design. The first study day was scheduled within 28 days after the screening examination. Subjects had to abstain from intake of alcohol and stimulating beverages containing xanthine derivates (tea, coffee, etc.) 12 hours before the study day. At the beginning of the study day, a pregnancy test was performed in females of childbearing potential. After the instillation of one drop of tropicamide 0.5% (Mydriaticum “Agepha” 0.5%, Vienna, Austria), a resting period of 20 minutes was scheduled. Thereafter, baseline measurements of retinal oxygen saturation using the oxygen module of the dynamic vessel analyzer (DVA) and retinal blood flow using LSFG were performed. During breathing of ambient room air the procedures were performed twice to obtain data for reproducibility analysis. In addition, capillary blood samples for evaluation of systemic oxygen saturation and partial pressures of CO2 (pCO2) and O2 (pO2) were drawn out of the arterialized earlobe.

Afterwards, a 30 minutes breathing period with either 100% oxygen or 12% oxygen in nitrogen was scheduled. A face mask covering mouth and nose, connected to a two-valve system that prevented the subject from rebreathing, was used to deliver gas to the subject. A randomization list determined whether study participants received oxygen or the oxygen-nitrogen mixture on the first or second day of the study. To ensure masking during the measurements, gas containers were covered with a black plastic foil. During the last 15 minutes of the breathing period retinal oximetry (DVA), LSFG and capillary blood sampling were repeated.

Heart rate and peripheral oxygen saturation were monitored continuously, blood pressure was measured every 5 minutes during the breathing period.

The second study day was scheduled within 14 days after the first study day. For baseline measurements and the breathing period the same schedule was performed but with the other gas mixture, according to the randomization list.

### Gas Mixtures

Oxygen: SAUERSTOFF medizinisch, Messer GmbH, Industriestrasse 5, 2352 Gumpoldskirchen, Austria. Dose: 100%, breathing for a maximum of 30 min.

Nitrogen: STICKSTOFF medizinisch, Messer GmbH, Industriestrasse 5, 2352 Gumpoldskirchen, Austria. Dose: 88% nitrogen in 12% oxygen, breathing for a maximum of 30 min.

## Methods

### Noninvasive Measurement of Systemic Hemodynamics

Using an automated oscillometric device (Infinity Delta, Draeger, Luebeck, Germany) on the upper arm, systolic, diastolic and mean arterial blood pressure (SBP, DBP, MAP) were measured. Pulse rate and systemic oxygen saturation were recorded with the same device automatically by a finger pulse oximeter.

### IOP

IOP was measured using a Goldmann applanation tonometer mounted on the slit lamp. A drop of oxybuprocaine hydrochloride combined with sodium fluorescein was applied for local anesthesia to the cornea before each measurement.

### Oxygen Saturation and Retinal Vessel Diameter

Measurement of retinal oxygen saturation and accurate determination of retinal vessel diameters was performed with a commercially available Dynamic Vessel Analyzer (DVA; Imedos, Jena, Germany).^29^^,^^30^ The ability to measure oxygen saturation in retinal vessels is based on the different absorption characteristics of oxygenated and nonoxygenated hemoglobin. Two monochromatic fundus images are recorded simultaneously using a fundus camera (FF450; Carl Zeiss Meditec AG, Jena, Germany). The isosbestic wavelength applied in this system is 548 nm, while the oxygen-sensitive wavelength is 610 nm. At the isosbestic wavelength, oxygenated and nonoxygenated hemoglobin show the same absorption characteristics, whereas oxygenated hemoglobin becomes nearly transparent when illuminated at the oxygen-sensitive wavelength.^27^

After acquiring the fundus image, the examiner defined a circular region of the optic nerve head (ONH). Two additional circles, at a predefined distance, were automatically centered by the software around the ONH. The arteries and veins of interest were marked manually by the operator with a simple mouse click. The vessels were traced automatically, because the photometric edges of a vessel wall are located in the vicinity of the mouse cursor in the green channel image. Once edges have been identified, three or more edge segments were determined to trace the direction of the vessel. For each segment of the vessel, with a length of 3 to 10 pixels, 3 pixels on each side of the vessel, and all pixels inside the vessel, were considered. The pixels representing the vessel wall were not included. The grayscale values of the pixels inside and outside the vessel in the red and green camera channels were averaged and used to calculate oxygen saturation. Finally, the average oxygen saturation (SaO_2_) values were measured across each selected vessel. Additionally, the software provides vessel diameters that allow calculation of the radius of the vessels, which is required for calculation of retinal oxygen extraction.^29^^,^^31^^–^^33^

### Retinal Blood Flow

For measurement of retinal blood flow, a commercially available laser speckle flowgraph (LSFG; Nidek, Tokyo, Japan) was used. The LSFG consists of a fundus camera that is equipped with a diode laser (wavelength 830 nm) and a digital charge-coupled device camera (750 × 360 pixels). The underlying technology of the LSFG is based on the phenomenon that a rough surface illuminated by coherent light, such as a laser, generates a so called “speckle pattern.” Moving erythrocytes in retinal blood vessels create a variation in this speckle pattern, which can be described statistically. According to the movement of blood cells, the speckle pattern varies depending on the blood flow velocity. The main output parameter, measured by the LSFG analysis software (LSFG Analyzer, Version 1.0.1.1) is the mean blur rate (MBR), which represents relative blood flow velocity in arbitrary units (a.u.).^34^^–^^36^ One hundred eighteen images are captured continuously at a rate of 30 frames per second over a measurement period of four seconds. The LSFG analysis software automatically identifies the start and the end of a cardiac cycle recorded within these four seconds. Images showing the same phases of the cardiac cycle are combined into an image sequence that represents a complete cardiac cycle. The average signal intensity in each of the 750 × 360 pixels over the entire cardiac cycle is calculated to create a “composite map” showing the distribution of mean blood flow during one cardiac cycle in the ocular fundus. In this color-coded map, the area around the ONH is manually delineated with an “ellipsoid rubber band.” The software automatically detects vascular and tissue areas within the ONH area and calculates the MBR for the entire ONH area (MA), for the large vessels within the ONH (MV), and for the tissue area containing microvessels (MT).^34^^,^^37^^–^^40^

### Retinal Vessel Segment Selection

**Figure 1.**
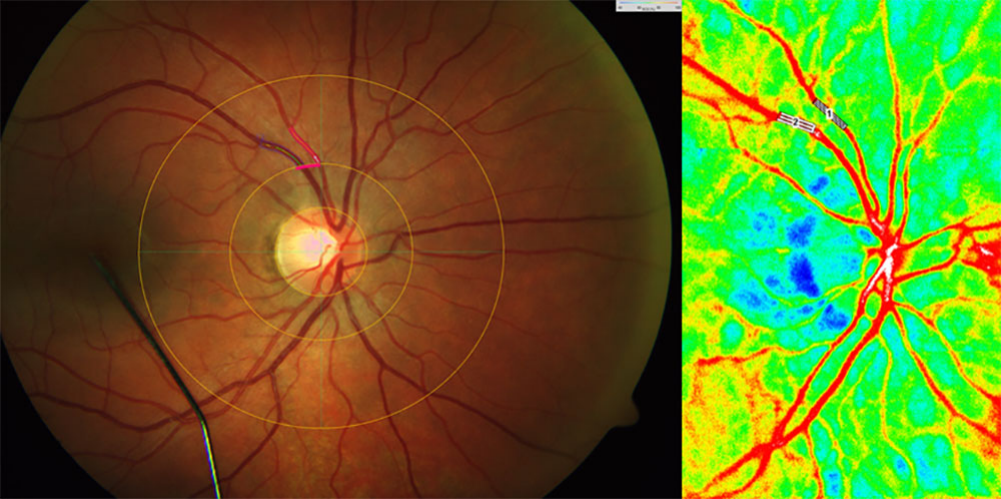
Sample image of the DVA measurement (*left panel*) and LSFG measurement (*right panel*). For both measurements, the same position at each vessel location was used. In both pictures, one artery and one vein are marked at the same location so that oxygen saturation and MBR can be obtained at the same vessel segment.

For calculation of retinal oxygen extraction, arteries and veins were first selected by the operator on the DVA image. Afterwards, the same vessels were selected for measuring ocular blood flow on the LSFG image. For determination of MBR in retinal arteries and veins, a “rectangular rubber band” was placed on the corresponding vessels in the composite map. After selection of the defined vessel, the average MBR was processed as previously published and used for calculation of retinal oxygen extraction, representing the value “Q” (as used in Formula 1 and 5).^19^ A sample image of how measurements were obtained is provided in Figure 1.

### Calculation of Retinal Oxygen Extraction

The formulas previously published by Werkmeister et al.^11^ were used to calculate retinal oxygen extraction with some modifications. As depicted in Figure 1 measurements were performed at the fundus, at some distance from the ONH, and not at the level of the central retinal artery (CRA) and vein (CRV). Therefore oxygen saturation values were corrected for the loss of oxygen (S_in_ – S_out_) across the arterial or venous wall between the measurement point and the point at which the vessels merge, with Q being the blood flow for each vessel obtained by LSFG instead as from Doppler OCT in the original publication. *R* is the radius of the vessel, and *L* is the distance between the vessel origin at the ONH and the point of measurement closest to the ONH. The radius *R* and the distance between the vessel origin *L* were both obtained by the DVA. *Hb* is the hemoglobin concentration and ${\bar{JO}}_{2}$ (oxygen flux) is the oxygen loss through the vascular wall:

(1) $$Q\cdot 1.35\cdot \mathrm{Hb}\cdot \left(S_{\mathrm{in}}-S_{\mathrm{out}}\right)=-2\pi \cdot R\cdot L\cdot {\bar{\mathrm{JO}}}_{2}$$

To calculate corrected oxygen saturation values (*cSaO_2_*), S_in_ − S_out_ was added to the measured SaO_2_ values in arteries, and for veins, S_in_ − S_out_ was subtracted from the measured SaO_2_ values.

(2) $$\mathrm{cSa}O_{2,A}=\mathrm{Sa}O_{2}+\left(S_{\mathrm{in}}-S_{\mathrm{out}}\right)$$

(3) $$\mathrm{cSa}O_{2,V}=\mathrm{Sa}O_{2}-\left(S_{\mathrm{in}}-S_{\mathrm{out}}\right)$$

To calculate oxygen saturation at the level of the central retinal artery (*SaO_2,CRA_*), the corrected oxygen saturations for all arteries (*#A*) obtained with formula (2) were used:

(4) $$\mathrm{Sa}O_{2,\mathrm{CRA}}=\frac{1}{\#A}\sum_{i=1}^{\#A}{\left(\mathrm{cSa}O_{2,A}\right)}_{i}$$

For retinal veins, blood flow had to be considered to calculate the oxygen saturation at the level of the central retinal vein (*SaO_2,CRV_*). The corrected oxygen saturation for each vein (*cSaO_2_,_V_*) was multiplied with the blood flow in each vein *Q_V,j_* and then divided by total blood flow in all veins *Q_V,tot_* (calculated as the sum of MBR in all veins).

(5) $$\mathrm{Sa}O_{2,\mathrm{CRV}}=\frac{1}{Q_{V,\mathrm{tot}}}\sum_{j=1}^{\#V}{\left(\mathrm{cSa}O_{2,V}\right)}_{j}\cdot Q_{V,j}$$

The following formulas were used to calculate corrected oxygen content at the level of the central retinal artery and vein (*cO_2,CRA_* and *cO_2,CRV_*).

(6) $$cO_{2,\mathrm{CRA}}=1.35\cdot \mathrm{Hb}\cdot \mathrm{Sa}O_{2,\mathrm{CRA}}+0.003\cdot PO_{2,\mathrm{CRA}}$$

(7) $$cO_{2,\mathrm{CRV}}=1.35\cdot \mathrm{Hb}\cdot \mathrm{Sa}O_{2,\mathrm{CRV}}+0.003\cdot PO_{2,\mathrm{CRV}}$$

Finally, retinal oxygen extraction (*extO_2_*) was calculated by subtracting the corrected venous from the arterial (arteriovenous difference in corrected oxygen content) oxygen content and by multiplying it with mean vessel flow rate (*MV*):

(8) $$\mathrm{ext}O_{2}=\left(cO_{2,\mathrm{CRA}}-cO_{2,\mathrm{CRV}}\right)\cdot \mathrm{MV}$$

### Statistical Analysis

Statistical analysis was performed using IBM SPSS Statistics (Version 28; IBM, Armonk, New York, USA). All values are presented as means ± SD. Normal distribution for all major outcome variables was confirmed using the Shapiro-Wilk test. Descriptive statistics are reported for all values obtained. For all major outcome variables, an analysis of variance (ANOVA) model was created comparing the two study days (hyperoxia and hypoxia). Contrasts were calculated between baseline values and values obtained during gas breathing. Percent changes between baseline values and during gas breathing were also calculated for the main outcome variables. A *P* value < 0.05 was considered as the level of significance.

For short-term repeatability analysis, data obtained from the baseline measurements on the same study day within 30 minutes were used, while for long-term reproducibility baseline data from the two study days were compared. To quantify reliability of measurements, intraclass correlation coefficients (ICC) were calculated with values less than 0.5 representing poor, between 0.5 and 0.75 representing moderate, between 0.75 and 0.90 representing good, and greater than 0.90 representing excellent repeatability.^41^ In addition, the coefficient of variation (CoV) was calculated. Figures were created using Graph Pad Prism (Version 10.3.0; GraphPad Software, San Diego, CA, USA).

## Results

A total of 22 subjects participated in the present study, of which 12 were female and 10 were male. Mean age was 27 ± 3 years, and all subjects were healthy and did not take any concomitant medication except for some women who were on hormonal contraception. Eighteen participants also participated in the repeatability and reproducibility experiments (11 female and seven male). One subject was excluded from analysis of the hyperoxia part, because the quality of the fundus photograph for measurement of oxygen saturation was not sufficient.

### ANOVA Model

**Figure 2.**
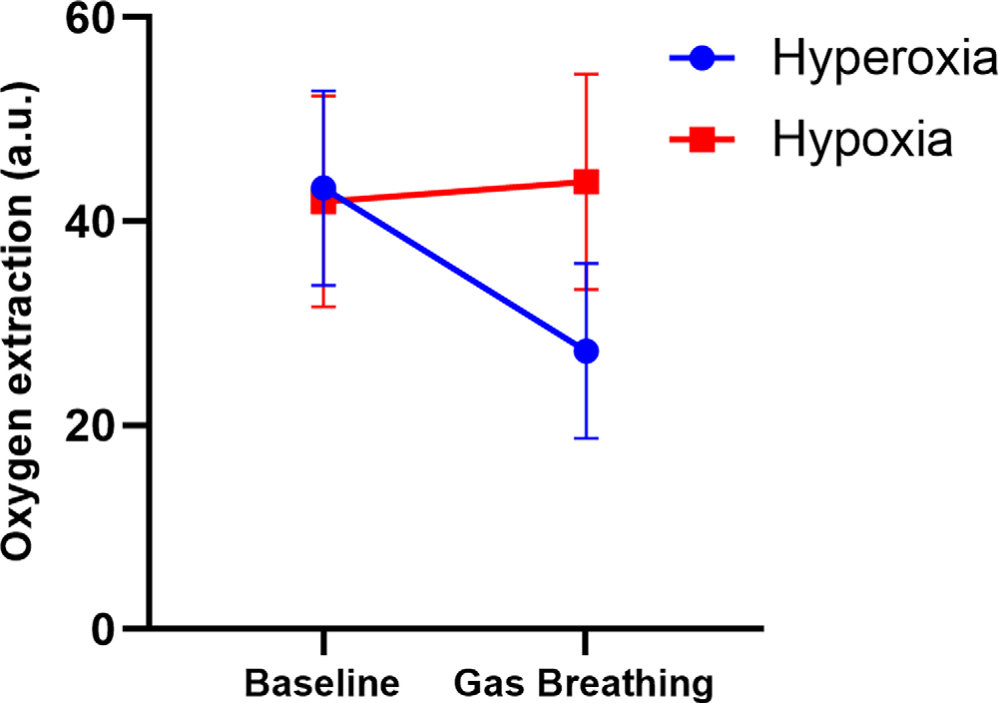
Retinal oxygen extraction during hyperoxia (*blue line*, n = 21) and hypoxia (*red line*, n = 22). Data are presented as means ± SD.

As shown in Table 1, there was a significant difference in the time course for heart rate, sO_2_ and pO_2_ between the two study days, whereas no difference for systolic blood pressure, diastolic blood pressure, mean arterial pressure or pCO_2_ was found. For retinal oxygen extraction, a significant difference was found between the two study days (*P* < 0.001, Fig. 2). Results for all other outcome parameters within the ANOVA model are presented in Table 2.

**Table 1.** Systemic Parameters at Baseline and During Gas Breathing on Both Study Days

	Hyperoxia (n = 21)			Hypoxia (n = 22)			
	Baseline	100% Oxygen	*P* Value	Baseline	88% Nitrogen	*P* Value	ANOVA Model *P* Value
Systolic blood pressure (mm Hg)	120 ± 13	119 ± 12	0.375	119 ± 14	117 ± 13	0.256	0.829
Diastolic blood pressure (mm Hg)	73 ± 10	76 ± 7	0.184	75 ± 10	76 ± 9	0.741	0.378
Mean arterial blood pressure (mm Hg)	92 ± 10	92 ± 7	0.797	92 ± 10	92 ± 9	0.918	0.915
Heart rate (bpm)	70 ± 10	63 ± 9	<0.001	72 ± 12	74 ± 13	0.319	0.001
sO_2_ (%)	97.4 ± 0.9	99.7 ± 0.2	<0.001	97.6 ± 0.8	90.3 ± 3.6	<0.001	<0.001
pO_2_ (mm Hg)	95 ± 8	338 ± 44	<0.001	96 ± 15	55 ± 4	<0.001	0.001
pCO_2_ (mm Hg)	36 ± 4	35 ± 4	0.371	37 ± 4	36 ± 3	0.65	0.917

*P* values between baseline and gas breathing were calculated as contrasts within the ANOVA model. Values are presented as mean ± SD.

**Table 2.** Ocular Parameters at Baseline and During Gas Breathing on Both Study Days

	Hyperoxia (n = 21)			Hypoxia (n = 22)			
	Baseline	100% Oxygen	*P* Value	Baseline	88% Nitrogen	*P* Value	ANOVA model *P* Value
Retinal oxygen extraction (a.u.)	43 ± 10	27 ± 9	<0.001	42 ± 10	44 ± 11	0.153	<0.001
cO_2,CRA_ (%)	96 ± 4	97 ± 4	0.291	96 ± 3	94 ± 5	0.003	0.003
cO_2,CRV_ (%)	64 ± 8	69 ± 8	0.001	66 ± 6	65 ± 7	0.281	<0.001
MV (a.u.)	71 ± 11	52 ± 9	<0.001	73 ± 9	79 ± 8	< 0.001	<0.001
MT (a.u.)	25 ± 5	20 ± 4	<0.001	26 ± 5	29 ± 5	<0.001	<0.001
MA (a.u.)	38 ± 6	28 ± 4	<0.001	38 ± 6	42 ± 6	<0.001	<0.001

cO_2,CRA_, corrected arterial oxygen content; cO_2,CRV_, corrected venous oxygen content; MA, mean area flow rate; MT, mean tissue flow rate; MV, mean vessel flow rate.

*P* values between baseline and gas breathing were calculated as contrasts within the ANOVA model. Values are presented as mean ± SD.

### Hyperoxia

During 100% oxygen breathing, no changes in systemic hemodynamics such as systolic, diastolic and mean arterial blood pressure were observed, while heart rate significantly decreased (*P* < 0.001). As expected, oxygen saturation obtained by finger pulse oximeter (sO_2_) and pO_2_ increased significantly (*P* < 0.001) while almost no change in pCO_2_ occurred. All systemic parameters at baseline and during oxygen breathing are provided in Table 1.

**Figure 3.**
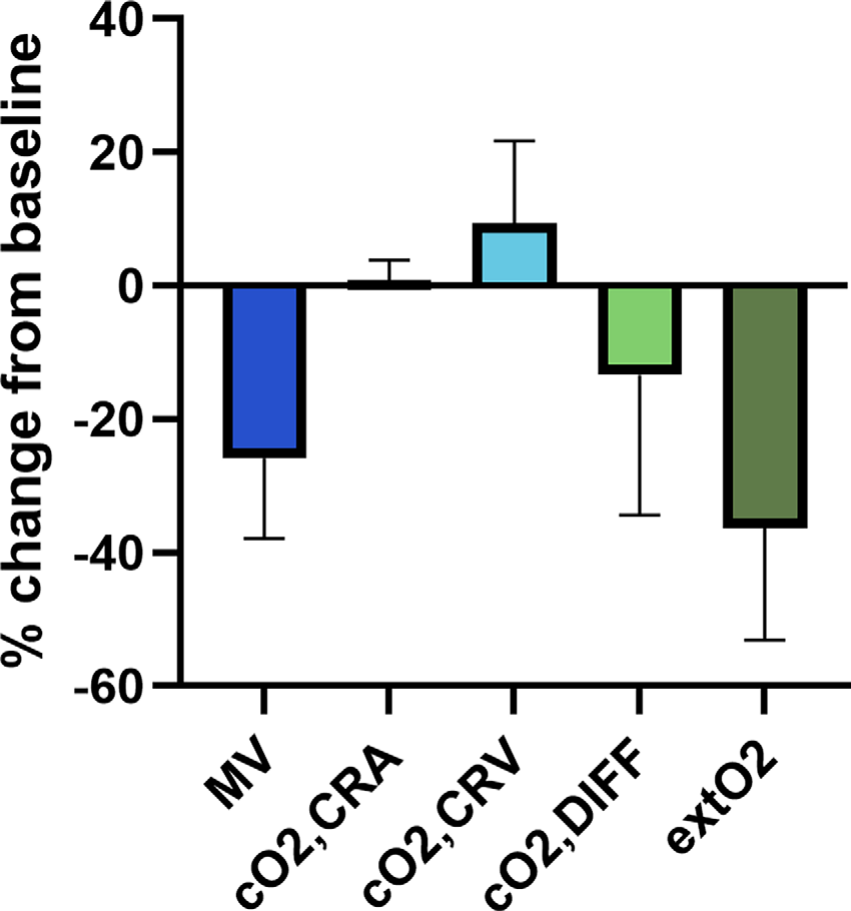
Percent change in mean vessel flow rate (MV), corrected arterial oxygen content (cO_2,CRA_), corrected venous oxygen content (cO_2,CRV_), arteriovenous difference in oxygen content (cO_2,DIFF_) and retinal oxygen extraction (extO_2_) in response to 100% oxygen breathing (n = 21). Data are presented as means ± SD.

In Figure 3, percent changes in retinal parameters during oxygen breathing are depicted. As expected, MV significantly decreased by 26% ± 12% from 71 ± 11 to 52 ± 9 a.u. (*P* < 0.001). Corrected oxygen saturation in the CRA only slightly increased from 96% ± 4% to 97% ± 4% (*P* = 0.291), whereas in the CRV a significant increase from 64% ± 8% to 69% ± 8% (*P* = 0.001) was observed. Correspondingly, the arteriovenous difference in corrected oxygen content decreased by 13% ± 21% (*P* = 0.004).

Because of the decrease in both, MV and arteriovenous difference in corrected oxygen content, a significant decrease in retinal oxygen extraction of 36% ± 17% from 43 ± 10 to 27 ± 9 a.u. (*P* < 0.001) was observed. Additionally, significant declines in MA and MT were observed during 100% oxygen breathing, MT decreased from 25 ± 5 to 20 ± 4 a.u. (*P* < 0.001) and MA decreased from 38 ± 6 to 28 ± 4 a.u. (*P* < 0.001).

### Hypoxia

During hypoxia, no changes in systemic hemodynamics or pCO2 occurred (*P* > 0.106 each). A pronounced decrease in sO_2_ and pO_2_ was observed (*P* < 0.001 each, Table 1).

**Figure 4.**
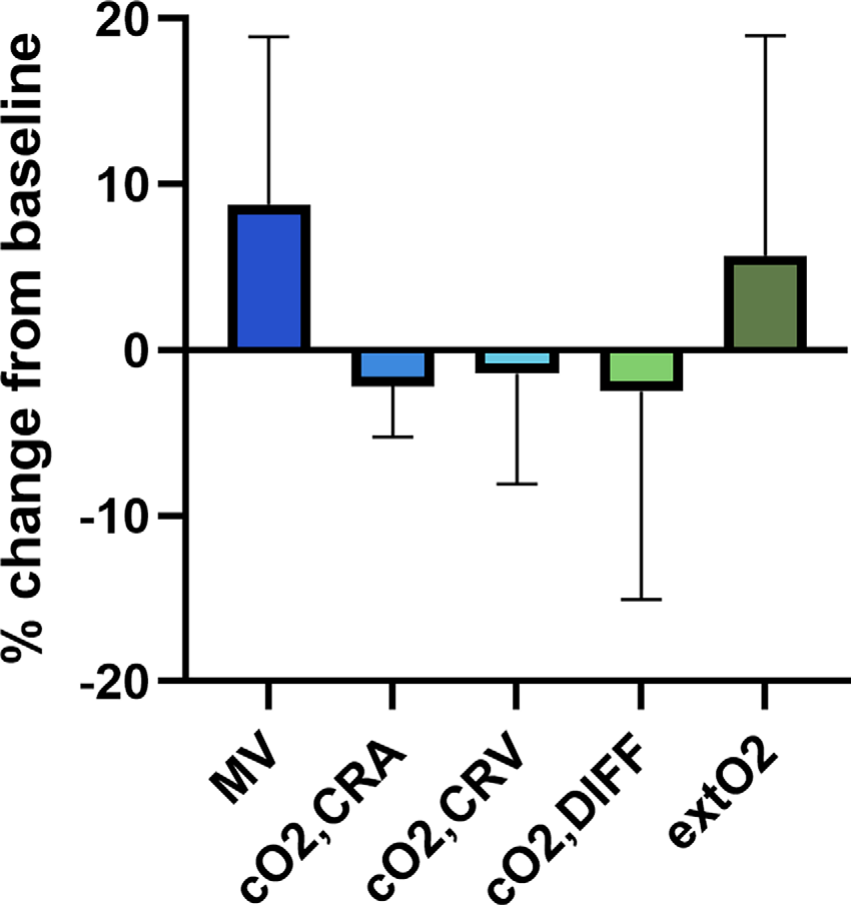
Percent change in mean vessel flow rate (MV), corrected arterial oxygen content (cO_2,CRA_), corrected venous oxygen content (cO_2,CRV_), arteriovenous difference in oxygen content (cO_2,DIFF_) and retinal oxygen extraction (extO_2_) in response to 88% nitrogen breathing (n = 22). Data are presented as means ± SD.

MV significantly increased by 9% ± 10% from 73 ± 9 to 79 ± 8 a.u. (*P* < 0.001). Corrected oxygen saturation in the CRA decreased from 96% ± 3% to 94% ± 5% (*P* = 0.003), whereas corrected oxygen saturation at the CRV remained stable (66% ± 6% vs. 65% ± 7%, *P* = 0.281). The arteriovenous difference in corrected oxygen content decreased by 2% ± 13%. Retinal oxygen extraction remained stable (42 ± 10 a.u. at baseline vs. 44 ± 11 a.u. during hypoxia, *P* = 0.153, Fig. 4). MT and MA increased from 26 ± 5 to 29 ± 5 a.u. (*P* < 0.001) and from 38 ± 6 to 42 ± 6 a.u. (*P* < 0.001), respectively.

### Reproducibility

**Figure 5.**
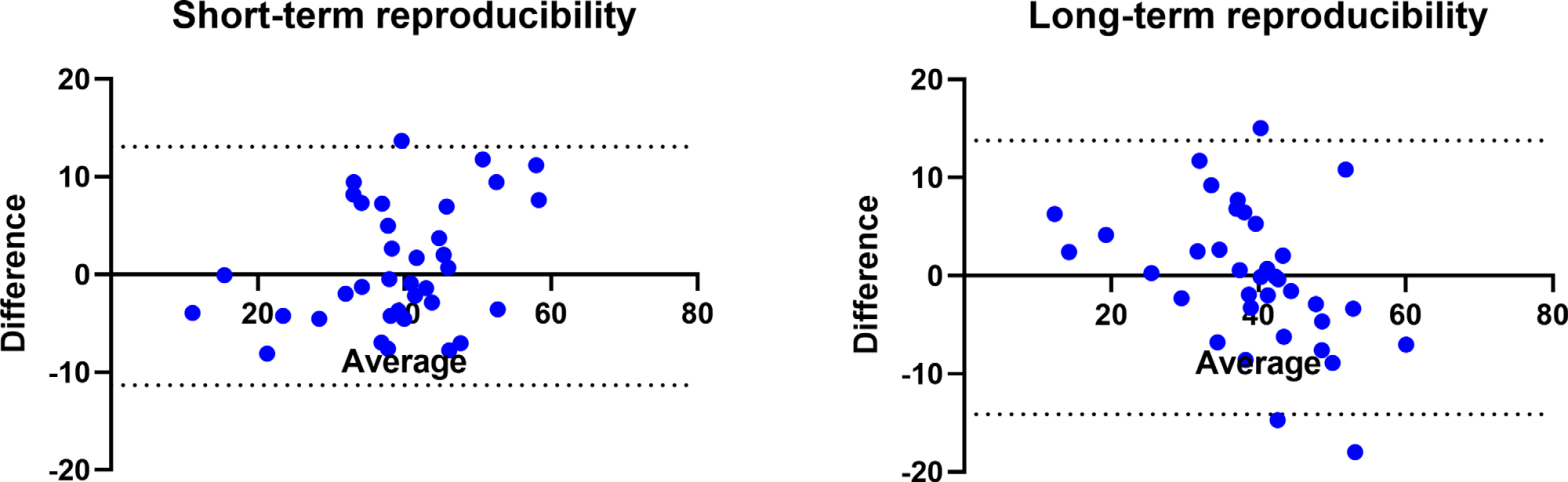
Bland-Altman plots for reproducibility data of oxygen extraction. The x-axis shows the mean between the two measurements (average), the y-axis shows the difference between the two measurements (difference). The data for short-term reproducibility are presented on the left side and the data for long-term reproducibility are presented on the right side. The mean value of the difference (*solid line*) and the 95% confidence intervals are also shown (*dashed line*).

Short-term reproducibility of retinal oxygen extraction measurements was excellent with an ICC of 0.910. The CoV between measurements was 9.8% ± 7.0%. For long-term reproducibility ICC was still good, although slightly lower (0.897). CoV between measurements was 10.4% ± 8.8%. Blant-Altmann plots for short- and long-term reproducibility are provided in Figure 5.

## Discussion

In the present study, we measured retinal oxygen extraction during breathing of 100% oxygen. Several previous human and animal experiments have found that during hyperoxia, retinal blood flow decreases while choroidal blood flow almost does not change. This leads to more oxygen being delivered to the inner layers of the retina originating from the choroid whereas retinal blood flow is reduced to avoid an oversupply with oxygen to the inner retina.^11^^,^^27^^,^^40^^,^^42^^–^^44^ In the present study, retinal oxygen extraction significantly decreased by approximately 36%. This is in line with previous publications using different set-ups, although the decrease observed in the present study was noticeably lower than reported in the literature. Palkovits et al.^27^ investigated retinal oxygen extraction during breathing of 100% oxygen and found a decrease in calculated oxygen extraction by 62.5%. In contrast to the present approach, retinal blood flow was measured using laser Doppler velocimetry (LDV) and blood flow values stem from one vein only, which is not representative for total blood flow. As described above, we obtained blood flow and oxygen saturation values from all retinal vessels around the optic nerve head in the present study. This was also achieved previously using Doppler OCT for measurement of blood flow in each larger retinal branch vessel, which resulted in a decrease in retinal oxygen extraction of approximately 55%.^11^ A possible explanation for the less-pronounced decrease in oxygen extraction found in the present study could lie within the technique of LSFG itself; because a saturation effect has been reported previously and especially at higher blood flow values, the correlation between velocity and MBR is not linear.^19^ Even though the estimation of retinal oxygen extraction with the current technique may have some limitations, it could still represent an easily accessible biomarker for retinal hypoxia.

As a second step, we measured retinal oxygen extraction during hypoxia. As for hyperoxia, our results again are in line with the literature, showing no change in retinal oxygen extraction during systemic hypoxia.^28^^,^^45^ This can be physiologically explained by the fact that in healthy individuals, the systemic drop in oxygen saturation is compensated by an increase in blood flow as shown in various species including humans. Although in healthy subjects blood flow can compensate for the drop in systemic oxygen saturation, this might not be the case in patients, as has been shown for example for patients with type I diabetes.^46^

When looking at the reproducibility of our method, it was found to be excellent in the short term and still good in the long-term. The ICCs obtained for retinal oxygen extraction in our study are similar to those reported for Doppler OCT.^15^^,^^47^ Because there is no generally accepted method for measurement of retinal oxygen extraction, no reproducibility data have been published previously.

The present approach has several strengths but also some limitations. Most previously published approaches for measurement of retinal oxygen extraction rely on blood flow measurements obtained by not commercially available methods such as Doppler-OCT or LDV. As stated above, Doppler-OCT provides reliable results for all major retinal arteries and veins, but measurements are time consuming and exhausting for patients. LDV shares the disadvantage of lengthy data acquisition and multiple retinal vessels cannot be measured simultaneously. By contrast, LSFG has the advantage that measurements only take approximately four seconds. Recently, approaches to calculate retinal oxygen extraction from retinal oximetry combined with diameter measurements or OCT-angiography (OCT-A) have been published.^48^^,^^49^ However, as for MBR in LSFG, the relation between decorrelation and velocity in OCT-A is not linear.^50^^,^^51^

As stated previously, another method that is used frequently in several publications is to estimate retinal oxygen extraction simply by calculating the difference between arterial and venous oxygen saturation.^52^^,^^53^ However, this approach does not take blood flow into account and could therefore lead to wrong conclusions regarding retinal oxygen extraction, as can be seen when looking at our results. During hypoxia, arteriovenous difference in oxygen saturation decreased. Without taking retinal blood flow into account, this could be interpreted as a decline in retinal oxygen extraction. Because blood flow increases in parallel, this does not reflect a change in retinal oxygen extraction.

Weaknesses of our approach include that in contrast to the above-mentioned methods, LSFG only provides blood flow values as arbitrary units and not as µL/min for example. Therefore further experiments are necessary to confirm whether the method can detect differences between and not only within subjects.^1^^–^^3^

A recent publication suggests that systemic blood pressure might have an influence on retinal oxygen extraction. We did not correct for blood pressure in the present studies, however, because our study population was generally healthy with blood pressure values within the normal range, we deem it unlikely that this could have had an influence on our measurements.^49^

Finally, we did not compare our approach to other measurement techniques, such as the combination of Doppler-OCT or LDV with retinal oximetry that have been published previously.^11^^,^^27^^,^^28^ However, because no gold standard for the measurement of retinal oxygen extraction exists, there is no ideal comparator.

In summary, we present a new approach for measurement of retinal oxygen extraction based on LSFG and retinal oximetry that shows plausible results and an excellent short-term and good long-term reproducibility in healthy subjects. To further evaluate the method, studies in patient groups are necessary. In the future, the technique might be used in longitudinal studies and because of the availability of the devices, multicenter studies could be an option as well.
